# Supplementary material for: Effective Halogen-Free Flame-Retardant Additives for Crosslinked Rigid Polyisocyanurate Foams: Comparison of Chemical Structures
Source: Materials (Basel). 2022 Dec 24;16(1):172. doi: 10.3390/ma16010172 (PMC9821766; doi:10.3390/ma16010172)
Supplement: Supplementary file 1 [file materials-16-00172-s001.zip › materials-2080238-supplementary.pdf]

Effective halogen-free flame retardant additives for crosslinked rigid polyisocyanurate foams: comparison of chemical structures

Johannes Lenz [1], Doris Pospiech [1,*], Hartmut Komber [1], Andreas Korwitz [1], Oliver Kobsch [1], Maxime Paven [2], Rolf W. Albach [2], Martin Günther [3], Bernhard Schartel [3]

[1] Leibniz-Institut für Polymerforschung Dresden e.V., Hohe Str. 6, 01069 Dresden, Germany
[2] Covestro Deutschland AG, 51365 Leverkusen, Germany
[3] Bundesanstalt für Materialforschung und-prüfung (BAM), 12205 Berlin, Germany

*** Correspondence pospiech@ipfdd.de; Tel.: (+49-351-4658497)**

Supplementary Materials

Content:

1. **^{1}H, ^{13}C and ^{31}P NMR data of phospha-aldol adducts**
2. **^{1}H, ^{13}C and ^{31}P NMR spectra of phospha-aldol adducts**
3. **PIR foam compositions studied**
4. **Compression strength of PIR foams with phospha-aldol adducts**
5. **Thermal decomposition of the PIR foams studied by TGA and Py-GC/MS**
6. **Cone calorimeter results: MARHE vs. char plots**

1. ^{1}H, ^{13}C and ^{31}P NMR data

FA-BPPO

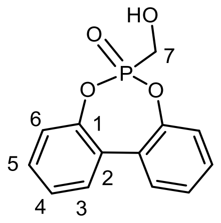

Figure S1. Chemical structure of **FA-BPPO.**

^{1}H NMR (500 MHz, DMSO-d$_6$): δ 7.67 (d, J = 7.8 Hz, 2H; 3), 7.54 (t, J = 7.8 Hz, 2H; 5), 7.45 (t, J = 7.5 Hz, 2H; 4), 7.37 (d, J = 8.1 Hz, 2H; 6), 5.79 (m, $^{3}J_{HH}$ = 5.8 Hz, $^{3}J_{PH}$ = 8.6 Hz, 1H; OH), 4.09 ppm (m, $^{3}J_{HH}$ = 5.8 Hz, $^{2}J_{PH}$ = 4.9 Hz, 2H; 7). ^{13}C NMR (125 MHz, DMSO-d$_6$): δ 147.6 (d, $^{2}J_{PC}$ = 10.5 Hz; 1), 130.3 (3 and 5), 128.1 (2), 126.6 (4), 121.7 (d, $^{3}J_{PC}$ = 3.7 Hz; 6), 54.6 ppm (d, $^{1}J_{PC}$ = 156 Hz; 7). ^{31}P NMR (202 MHz, DMSO-d$_6$): δ 33.7 ppm.

AA-BPPO

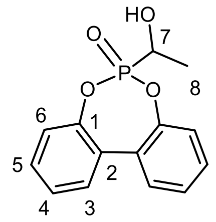

Figure S2. Chemical structure of **AA-BPPO.**

^{1}H NMR (500 MHz, DMSO-d$_6$): δ 7.65 (2H; 3), 7.52 (2H; 5), 7.43 (2H; 4), 7.37 (2H; 6), 5.91 (dd, $^{3}J_{HH}$ = 6.6 Hz, $^{3}J_{PH}$ = 8.5 Hz, 1H; OH), 4.25 (m, $^{3}J_{HH}$ = 6.8 Hz, $^{2}J_{PH}$ = 2.9 Hz, 1H; 7), 1.46 ppm (dd, $^{3}J_{HH}$ = 7.0 Hz, $^{3}J_{PH}$ = 18.7 Hz, 3H; 8). ^{13}C NMR (125 MHz, DMSO-d$_6$): δ 147.9 and 147.7 (2 x d, $^{2}J_{PC}$ = 10.6 Hz; 1), 130.3 (two signals; 3), 130.2 (5), 128.0 (two signals; 2), 126.5 and 126.4 (4), 121.8 and 121.7 (2 x d, $^{3}J_{PC}$ = 3.6 Hz; 6), 61.7 (d, $^{1}J_{PC}$ = 155 Hz; 7), 17.8 ppm (8). ^{31}P NMR (202 MHz, DMSO-d$_6$): δ 35.8 ppm. The chirality of C_7 results in non-equivalence of the protons and carbons in the biphenyl moiety. The proton signals of H_3 – H_6 show a complex pattern.

FU-BPPO

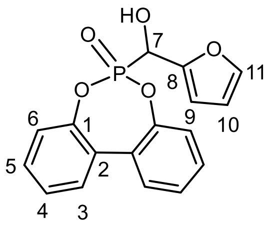

Figure S3. Chemical structure of **FU-BPPO**.

^{1}H NMR (500 MHz, DMSO-d$_6$): δ 7.71 (s, 1H; 11), 7.66 (2H; 3), 7.52 (2H; 5), 7.44 (2H; 4), 7.38 and 7.26 (2 x d, J = 8.1 Hz, 2 x 1H; 6), 6.69 (dd, $^{3}J_{HH}$ = 6.6 Hz, $^{3}J_{PH}$ = 13.9 Hz, 1H; OH), 6.62 (m, 1H; 9), 6.50 (m, 1H; 10), 5.32 ppm (dd, $^{3}J_{HH}$ = 6.6 Hz, $^{2}J_{PH}$ = 12.9 Hz, 1H; 7). ^{13}C NMR (125 MHz, DMSO-d$_6$): δ 149.6 (8), 148.0 and 147.5 (2 x d, $^{2}J_{PC}$ = 10.5 Hz; 1), 143.7 (d, $^{4}J_{PH}$ = 2.2 Hz; 11), 130.4 and 130.3 (3), 130.2 (5), 128.0 and 127.8 (2), 126.6 and 126.5 (4), 121.8 ($^{3}J_{PC}$ = 3.6 Hz; 6), 110.9 (10), 110.2 (d, $^{3}J_{PC}$ = 7.3 Hz; 9), 61.6 ppm (d, $^{1}J_{PC}$ = 165 Hz; 7). ^{31}P NMR (202 MHz, DMSO-d$_6$): δ 28.3 ppm.

The chirality of C_7 results in non-equivalence of the protons and carbons in the biphenyl moiety. The proton signals of H_3 – H_6 show a complex pattern.

BA-BPPO

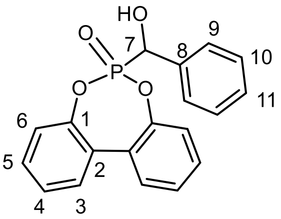

Figure S4. Chemical structure of **BA-BPPO**.

^{1}H NMR (500 MHz, DMSO-d_6): δ 7.65 (d, J = 7.7 Hz, 2H; 3), 7.6-7.48 (4H; 5, 9), 7.48-7.39 (4H; 4, 10), 7.39-7.3 (2H; 6_A, 11), 7.25 (d, 8.1 Hz, 1H; 6_B), 6.64 (dd, $^3J_{HH}$ = 5.9 Hz, $^3J_{PH}$ = 16.5 Hz, 1H; OH), 5.33 ppm (dd, $^3J_{HH}$ = 5.9 Hz, $^2J_{PH}$ = 11.3 Hz, 1H; 7). ^{13}C NMR (125 MHz, DMSO-d_6): δ 148.2 and 147.7 (2 x d, $^2J_{PC}$ = 10.4 Hz; 1), 136.7 (8), 130.4 (3), 130.3 and 130.2 (two signals; 5), 128.3 (10, 11), 128.0 and 127.9 (2), 127.7 (d, $^3J_{PC}$ = 6.3 Hz; 9), 126.5 and 126.4 (4), 121.9 and 121.8 (2 x d, $^3J_{PC}$ = 3.2 Hz; 6), 68.8 ppm (d, $^1J_{PC}$ = 156 Hz; 7). ^{31}P NMR (202 MHz, DMSO-d_6): δ 31.1 ppm. The chirality of C_7 results in non-equivalence of the protons and carbons in the biphenyl moiety. The proton signals of H_3 – H_6 show a complex pattern.

TA-BPPO

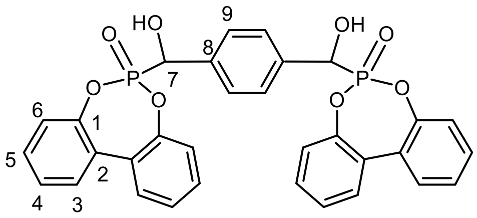

Figure S5. Chemical structure of **TA-BPPO**.

^{1}H NMR (500 MHz, DMSO-d_6): δ 7.67 (d, J = 7.7 Hz, 4H; 3), 7.61 (s, 4H; 9), 7.53 (4H; 5), 7.44 (4H; 4), 7.33 and 7.28 (2 x 1H; 6), 6.69 (dd, $^3J_{HH}$ = 5.8 Hz, $^3J_{PH}$ = 16.5 Hz, 2H; OH), 5.38 ppm (dd, $^3J_{HH}$ = 5.8 Hz, $^2J_{PH}$ = 2.3 Hz, 2H; 7). ^{13}C NMR (125 MHz, DMSO-d_6): δ 148.2 and 147.6 (1), 136.7 (8), 130.3 (two signals; 3, 5), 128.0 and 127.9 (2), 127.7 (9), 126.6 and 126.5 (4), 121.9 and 121.8 (6), 68.6 ppm (d, $^1J_{PC}$ = 156 Hz; 7). ^{31}P NMR (202 MHz, DMSO-d_6): δ 31.0 ppm. The chirality of both C_7 atoms results in a pair of diastereomers (RR/SS and RS/SR). Therefore, an additional signal splitting is observed both in the ^{1}H and ^{13}C NMR spectrum.

FA-DOPO

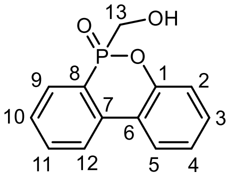

Figure S6. Chemical structure of **FA-DOPO.**

^{1}H NMR (500 MHz, DMSO-d$_6$): δ 8.25 (dd, $^{4}J_{PH}$ = 4.4 Hz, $^{3}J_{HH}$ = 7.8 Hz, 1H; 12), 8.19 (d, $^{3}J_{HH}$ = 8.0 Hz, 1H; 5), 7.96 (dd, $^{3}J_{HH}$ = 7.6 Hz, $^{3}J_{PH}$ = 12.7 Hz, 1H; 9), 7.80 (t, $^{3}J_{HH}$ = 7.8 Hz, 1H; 11), 7.45 (dt, $^{4}J_{PH}$ = 2.9, $^{4}J_{PH}$ = 7.3 Hz, 1H; 10), 7.46 (t, $^{3}J_{HH}$ = 7.6 Hz, 1H; 3), 7.31 (t, $^{3}J_{HH}$ = 7.8 Hz, 1H; 4), 7.46 (d, $^{3}J_{HH}$ = 8.2 Hz, 1H; 2), 5.56 (dt, $^{3}J_{PH}$ = 9.6 Hz, $^{3}J_{HH}$ = 6.0 Hz, 1H; OH), 4.27 (dd, $^{2}J_{HH}$ = 15.2 Hz, $^{3}J_{HH}$ = 6.0 Hz, 1H; one H of 13), 4.06 ppm (m, $^{2}J_{HH}$ = 15.2 Hz, $^{3}J_{HH}$ = 6.0 Hz, $^{2}J_{PH}$ = 8.2 Hz, 1H; one H of 13). ^{13}C NMR (125 MHz, DMSO-d$_6$): δ 149.1 (d, $^{2}J_{PC}$ = 9.2 Hz; 1), 135.3 (d, $^{2}J_{PC}$ = 4.8 Hz; 7), 133.4 (d, $^{4}J_{PC}$ = 1.8 Hz; 11), 130.7 (3), 130.5 (d, $^{2}J_{PC}$ = 12.2 Hz; 9), 128.6 (d, $^{3}J_{PC}$ = 13.1 Hz; 10), 125.6 (5), 124.5 (4), 124.0 (d, $^{3}J_{PC}$ = 8.8 Hz; 12), 123.2 (d, $^{1}J_{PC}$ = 114.1 Hz; 8), 121.4 (d, $^{3}J_{PC}$ = 10.4 Hz; 6), 120.0 (d, $^{3}J_{PC}$ = 5.8 Hz; 2), 57.8 ppm (d, $^{1}J_{PC}$ = 118.2 Hz; 13). ^{31}P NMR (202 MHz, DMSO-d$_6$): δ 33.4 ppm.

AA-DOPO

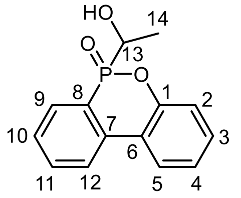

Figure S7. Chemical structure of **AA-DOPO**.

^{1}H NMR (500 MHz, DMSO-d$_6$): δ 8.22 (1H; 12), 8.16 (1H; 5), 7.93 (dd, $^{3}J_{HH}$ = 7.6 Hz, $^{3}J_{PH}$ = 12.2 Hz, 0.21H; 9$_{mi}$), 7.90 (dd, $^{3}J_{HH}$ = 7.6 Hz, $^{3}J_{PH}$ = 11.5 Hz, 0.79H; 9$_{mi}$), 7.80 (1H; 11), 7.60 (1H; 10), 7.43 (1H; 3), 7.33-7.22 (2H; 2,4), 5.61 (1H; OH), 4.35 (dq, $^{2}J_{PH}$ = 13.3 Hz, $^{3}J_{HH}$ = 6.8 Hz, 0.21H; 13$_{mi}$), 4.10 (m, 0.45H; 13$_{ma}$), 1.35 ppm (3H; 14). ^{13}C NMR (125 MHz, DMSO-d$_6$): δ 149.7 (d, $^{2}J_{PC}$ = 9.0 Hz; 1$_{ma}$), 149.5 (d, $^{2}J_{PC}$ = 9.6 Hz; 1$_{mi}$), 135.8 (d, $^{2}J_{PC}$ = 5.5 Hz; 7$_{ma}$), 135.7 (d, $^{2}J_{PC}$ = 4.8 Hz; 7$_{mi}$), 133.6 (d, $^{4}J_{PC}$ = 1.2 Hz; 11$_{ma}$), 133.3 (d, $^{4}J_{PC}$ = 1.2 Hz; 11$_{mi}$), 131.4 (d, $^{2}J_{PC}$ = 10.1 Hz; 9$_{ma}$), 130.7 (3$_{ma}$), 130.65 (d, $^{2}J_{PC}$ = 10.4 Hz; 9$_{mi}$), 130.6 (3$_{mi}$), 128.5 (d, $^{3}J_{PC}$ = 12.0 Hz; 10$_{mi}$), 128.4 (d, $^{3}J_{PC}$ = 12.1 Hz; 10$_{ma}$), 125.6 (5$_{ma}$), 125.3 (5$_{mi}$), 124.4 (4$_{ma}$), 124.3 (4$_{mi}$), 124.0 (d, $^{3}J_{PC}$ = 8.8 Hz; 12$_{ma}$), 123.8 (d, $^{3}J_{PC}$ = 9.0 Hz; 12$_{mi}$), 122.9 (d, $^{1}J_{PC}$ = 111.3 Hz; 8$_{mi}$), 122.7 (d, $^{1}J_{PC}$ = 109.8 Hz; 8$_{ma}$), 121.7 (d, $^{3}J_{PC}$ = 9.3 Hz; 6$_{ma}$), 121.4 (d, $^{3}J_{PC}$ = 9.7 Hz; 6$_{mi}$), 119.9 (d, $^{2}J_{PC}$ = 5.6 Hz; 2$_{mi}$), 119.7 (d, $^{2}J_{PC}$ = 5.9 Hz; 2$_{ma}$), 65.0 (d, $^{1}J_{PC}$ = 118.9 Hz; 13$_{ma}$), 64.1 (d, $^{1}J_{PC}$ = 119.6 Hz; 13$_{mi}$), 16.9 (d, $^{2}J_{PC}$ = 2.4 Hz; 14$_{ma}$), 16.5 ppm (14$_{mi}$). ^{31}P NMR (202 MHz, DMSO-d$_6$): δ 34.6 (P$_{mi}$) and 34.2 ppm (P$_{ma}$).
Note: The chirality of both the phosphorous atom and methine carbon C$_{13}$ results in two diastereomers with overlapping signals both in the ^{1}H and ^{13}C NMR spectrum. The molar ratio of major (ma) to minor (mi) isomer is 79 : 21.

FU-DOPO

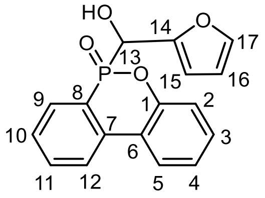

Figure S8. Chemical structure of **FU-DOPO**.

^{1}H NMR (500 MHz, DMSO-d$_6$): δ 8.22 (1H; 12), 8.15 (1H; 5), 7.93 (dd, $^{3}J_{HH}$ = 7.6 Hz, $^{3}J_{PH}$ = 12.5 Hz, 0.55H; 9$_{ma}$), 7.80 (1H; 11), 7.75 (dd, $^{3}J_{HH}$ = 7.7 Hz, $^{3}J_{PH}$ = 12.3 Hz, 0.45H; 9$_{mi}$), 7.65-7.55 (1.45H; 10,17$_{mi}$), 7.54 (s, 0.55H; 17$_{ma}$), 7.43 (1H; 3), 7.30-7.18 (2H; 2,4), 6.48-6.32 (3H; 15,16,OH), 5.30 (dd, $^{2}J_{PH}$ = 9.2 Hz, $^{3}J_{HH}$ = 6.2 Hz, 0.55H; 13$_{ma}$), 4.06 ppm (dd, $^{2}J_{PH}$ = 12.3 Hz, $^{3}J_{HH}$ = 6.2 Hz, 0.45H; 13$_{mi}$). ^{13}C NMR (125 MHz, DMSO-d$_6$): δ 150.2 (14), 149.8 (d, $^{2}J_{PC}$ = 9.0 Hz; 1$_{mi}$), 149.7 (d, $^{2}J_{PC}$ = 9.4 Hz; 1$_{ma}$), 143.2 (d, $^{5}J_{PC}$ = 2.2 Hz; 17$_{mi}$), 143.1 (d, $^{5}J_{PC}$ = 2.2 Hz; 17$_{ma}$), 136.1 (d, $^{2}J_{PC}$ = 6.0 Hz; 7$_{mi}$), 136.0 (d, $^{2}J_{PC}$ = 5.5 Hz; 7$_{ma}$), 133.8 (d, $^{4}J_{PC}$ = 1.5 Hz; 11$_{mi}$), 133.7 (d, $^{4}J_{PC}$ = 1.5 Hz; 11$_{ma}$), 131.6 (d, $^{2}J_{PC}$ = 10.4 Hz; 9$_{mi}$), 131.1 (d, $^{2}J_{PC}$ = 10.4 Hz; 9$_{ma}$), 130.7 (3$_{mi}$), 130.6 (3$_{ma}$), 128.5 (d, $^{3}J_{PC}$ = 12.6 Hz; 10$_{ma}$), 128.3 (d, $^{3}J_{PC}$ = 12.6 Hz; 10$_{mi}$), 125.5 (5$_{mi}$), 125.4

(5_{ma}), 124.5 (4_{mi}), 124.3 (4_{ma}), 123.9 (d, $^3J_{PC}$ = 9.3 Hz; 12_{mi}), 123.7 (d, $^3J_{PC}$ = 9.3 Hz; 12_{ma}), 122.5 (d, $^1J_{PC}$ = 114.9 Hz; 8_{ma}), 122.2 (d, $^1J_{PC}$ = 114.9 Hz; 8_{mi}), 121.5 (d, $^3J_{PC}$ = 9.8 Hz; 6_{mi}), 121.3 (d, $^3J_{PC}$ = 9.8 Hz; 6_{ma}), 119.7 (2), 110.7 (16), 109.6 (d, $^3J_{PC}$ = 6.1 Hz; 15_{ma}), 109.3 (d, $^3J_{PC}$ = 6.7 Hz; 15_{mi}), 66.5 (d, $^1J_{PC}$ = 122.3 Hz; 8_{mi}), 65.7 ppm (d, $^1J_{PC}$ = 122.3 Hz; 8_{ma}). ^{31}P NMR (202 MHz, DMSO-d_6): δ 29.2 (P_{mi}) and 29.1 ppm (P_{ma}).
Note: The chirality of both the phosphorous atom and methine carbon C_{13} results in two diastereomers with overlapping signals both in the ^{1}H and ^{13}C NMR spectrum. The molar ratio of major (ma) to minor (mi) isomer is 55 : 45.

BA-DOPO

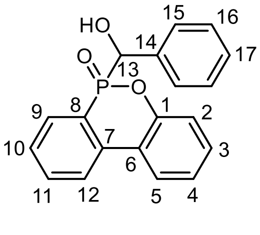

Figure S9. Chemical structure of **BA-DOPO**.

^{1}H NMR (500 MHz, DMSO-d_6): δ 8.20 (1H; 12), 8.15 (d, $^3J_{HH}$ = 7.8 Hz, 0.48H; 5_{mi}), 8.10 (d, $^3J_{HH}$ = 7.8 Hz, 0.52H; 5_{ma}), 7.96 (dd, $^3J_{PH}$ = 12.2 Hz, $^3J_{HH}$ = 7.5 Hz, 0.52H; 9_{ma}), 7.77 (1H; 11), 7.60 (dd, $^3J_{HH}$ = 7.4 Hz, $^4J_{PH}$ = 2.0 Hz, 0.52H; 10_{ma}), 7.52-7.15 (8.44H; 2_{mi},3,4,9_{mi},10_{mi}, 15,16,17), 7.12 (d, $^3J_{HH}$ = 7.9 Hz, 0.52H; 2_{ma}), 6.39 (dd, $^3J_{PH}$ = 17.3 Hz, $^3J_{HH}$ = 6.0 Hz, 0.48H; OH_{mi}), 6.30 (dd, $^3J_{PH}$ = 16.9 Hz, $^3J_{HH}$ = 5.5 Hz, 0.52H; OH_{ma}), 5.33 (m, 0.52H; 13_{ma}), 5.16 ppm (dd, $^3J_{PH}$ = 12.0 Hz, $^3J_{HH}$ = 6.0 Hz, 0.48H; 13_{mi}). ^{13}C NMR (125 MHz, DMSO-d_6): δ 150.2 (2 x d, $^2J_{PC}$ = 9.2 Hz; 1), 137.0 (14_{mi}), 136.9 (d, $^2J_{PC}$ = 2.5 Hz; 14_{ma}), 136.1 (7), 133.7 (d, $^4J_{PC}$ = 2.5 Hz; 11_{mi}), 133.5 (d, $^4J_{PC}$ = 2.0 Hz; 11_{ma}), 131.7 (d, $^2J_{PC}$ = 9.2 Hz; 9_{mi}), 131.0 (d, $^2J_{PC}$ = 10.6 Hz; 9_{ma}), 130.7 (3_{mi}), 130.5 (3_{ma}), 128.4 (d, $^3J_{PC}$ = 12.7 Hz; 10_{ma}), 128.0 (d, $^3J_{PC}$ = 12.2 Hz; 10_{mi}), 127.8-127.0 (several signals; 15,16,17), 125.5 (5_{mi}), 125.3 (5_{ma}), 124.3 (4_{mi}), 124.1 (4_{ma}), 123.8 (d, $^3J_{PC}$ = 9.1 Hz; 12_{mi}), 123.5 (d, $^3J_{PC}$ = 9.1 Hz; 12_{ma}), 122.8 (d, $^1J_{PC}$ = 112.8 Hz; 8_{ma}), 122.1 (d, $^1J_{PC}$ = 122.1 Hz; 8_{mi}), 121.6 (d, $^3J_{PC}$ = 9.2 Hz; 6_{mi}), 121.3 (d, $^3J_{PC}$ = 9.2 Hz; 6_{ma}), 119.7 (d, $^2J_{PC}$ = 6.2 Hz; 2_{mi}), 119.5 (d, $^2J_{PC}$ = 6.2 Hz; 2_{ma}), 71.9 (d, $^1J_{PC}$ = 116.0 Hz; 13_{mi}), 71.7 ppm (d, $^1J_{PC}$ = 116.0 Hz; 13_{ma}). ^{31}P NMR (202 MHz, DMSO-d_6): δ 31.0 and 30.9 ppm.
Note: The chirality of both the phosphorous atom and methine carbon C_{13} results in two diastereomers with overlapping signals both in the ^{1}H and ^{13}C NMR spectrum. The molar ratio of major (ma) to minor (mi) isomer is 52 : 48.

TA-DOPO

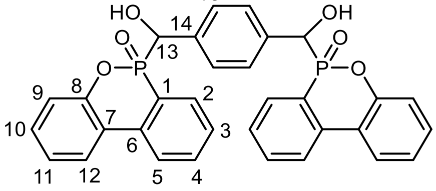

Figure S10. Chemical structure of **TA-DOPO**.

^{1}H NMR (500 MHz, DMSO-d_6): δ 8.28-8.20 (2H; 12), 8.20-8.12 (2H; 5), 8.02-7.93 (1.1H; 9_A), 7.85-7.75 (2H; 11), 7.65-7.55 (1.1H; 10_A), 7.50-7.34 (6H; 9_B,10_B,11,15_A), 7.34-7.24 (5.8H; 2,4,15_B), 6.45-6.20 (2H; OH), 5.39-5.31 (1.1H; 13_A), 5.22-5.10 ppm (0.9H; 13_B). ^{13}C NMR (125 MHz, DMSO-d_6): δ 150.1 (1), 136.7-136.2 (14), 136.1 (7), 133.7 (11_B), 133.5 (11_A), 131.7 (9_B), 130.9 (9_A), 130.7 (3_B), 130.6 (3_A), 128.5 (10_A), 128.0 (10_B), 127.0 (15_A), 126.6 (15_B), 125.6 (5_B), 125.4 (5_A), 124.4 (4_B), 124.2 (4_A), 124.0-123.5 (12), 123.5-121.5 (d, 8), 121.5-121.0 (6), 119.8-119.4 (2), 72.3-71.3 ppm (d, 13). ^{31}P NMR (202 MHz, DMSO-d_6): δ 31.0-30.2 ppm.

Note: The chirality of both phosphorous atoms and both methine carbon C_{13} results in several diastereomers with overlapping signals both in the ^{1}H and ^{13}C NMR spectrum.

FU-EP

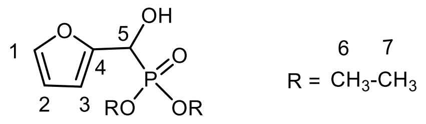

Figure S11. Chemical structure of **FU-EP**.

^{1}H NMR (500 MHz, DMSO-d_6): δ 7.64 (s, 1H; 1), 6.44 (2H; 2,3), 6.19 (dd, $^{3}J_{HH}$ = 6.5 Hz, $^{3}J_{PH}$ = 13.3 Hz, 1H; OH), 4.91 (dd, $^{3}J_{HH}$ = 6.5 Hz, $^{2}J_{PH}$ = 14.7 Hz, 1H; 5), 4.1-3.9 (4H; 6), 1.22 and 1.17 ppm (2 x t, $^{3}J_{HH}$ = 7.7 Hz, 2 x 3H; 7). ^{13}C NMR (125 MHz, DMSO-d_6): δ 151.5 (4), 142.7 (d, $^{4}J_{PC}$ = 2.2 Hz; 1), 110.7 (2), 108.7 (d, $^{3}J_{PC}$ = 6.4 Hz; 3), 63.4 (d, $^{1}J_{PC}$ = 170 Hz; 5), 62.4 and 62.1 (2 x d, $^{2}J_{PC}$ = 6.5 Hz; 6), 16.4 and 16.3 ppm (2 x d, $^{3}J_{PC}$ = 11.5 Hz; 7). ^{31}P NMR (202 MHz, DMSO-d_6): δ 19.6 ppm.

Note: The chirality of C_5 results in non-equivalence of the protons and carbons in the ethylester groups. The proton signals of H_6 show a complex pattern.

FU-BP

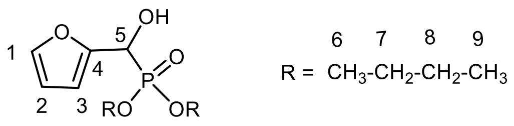

Figure S12. Chemical structure of **FU-BP**.

^{1}H NMR (500 MHz, DMSO-d_6): δ 7.63 (s, 1H; 1), 6.5-6.4 (2H; 2,3), 6.18 (dd, $^{3}J_{HH}$ = 6.5 Hz, $^{3}J_{PH}$ = 13.6 Hz, 1H; OH), 4.91 (dd, $^{3}J_{HH}$ = 6.5 Hz, $^{2}J_{PH}$ = 14.8 Hz, 1H; 5), 4.05-3.85 (4H; 6), 1.6-1.45 (4H; 7), 1.4-1.2 (4H; 8), 0.88 and 0.85 ppm (2 x t, $^{3}J_{HH}$ = 7.4 Hz, 2 x 3H; 9). ^{13}C NMR (125 MHz, DMSO-d_6): δ 151.6 (4), 142.7 (d, $^{4}J_{PC}$ = 2.2 Hz; 1), 110.6 (2), 108.7 (d, $^{3}J_{PC}$ = 6.1 Hz; 3), 65.9 and 65.6 (2 x d, $^{2}J_{PC}$ = 7.0 Hz; 6), 63.4 (d, $^{1}J_{PC}$ = 170 Hz; 5), 32.1 and 32.0 (2 x d, $^{3}J_{PC}$ = 10.8 Hz; 7), 18.2 (8), 13.4 ppm (9). ^{31}P NMR (202 MHz, DMSO-d_6): δ 19.6 ppm.

Note: The chirality of C_5 results in non-equivalence of the protons and carbons in the butylester groups. The proton signals of H_6-H_8 show a complex pattern.

TA-BP

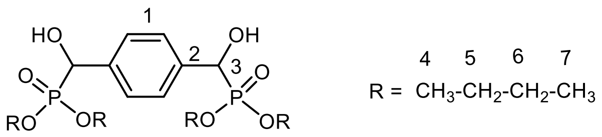

Figure S13. Chemical structure of **TA-BP**.

^{1}H NMR (500 MHz, DMSO-d_6): δ 7.63 (s, 4H; 1), 6.13 (dd, $^{3}J_{HH}$ = 5.8 Hz, $^{3}J_{PH}$ = 15.1 Hz, 2H; OH), 4.92 (dd, $^{3}J_{HH}$ = 5.8 Hz, $^{2}J_{PH}$ = 12.4 Hz, 2H; 3), 4.0-3.75 (8H; 4), 1.6-1.45 (8H; 5), 1.35-1.2 (8H; 6), 0.87 and 0.85 ppm (2 x t, $^{3}J_{HH}$ = 7.1 Hz, 2 x 6H; 7). ^{13}C NMR (125 MHz, DMSO-d_6): δ 137.7 (2), 126.7 (1), 69.3 and 69.2 (2 x d, $^{1}J_{PC}$ = 163 Hz; 3), 65.7 and 65.4 (2 x m; 4), 32.1 ($^{3}J_{PC}$ = 7.5 Hz; 5), 18.2 (6), 13.4 ppm (7). ^{31}P NMR (202 MHz, DMSO-d_6): δ 21.6 ppm.

Note: The chirality of C_3 results in non-equivalence of the protons and carbons in the butylester groups. The proton signals of H_4-H_6 show a complex pattern.

2. ^{1}H, ^{13}C and ^{31}P NMR spectra

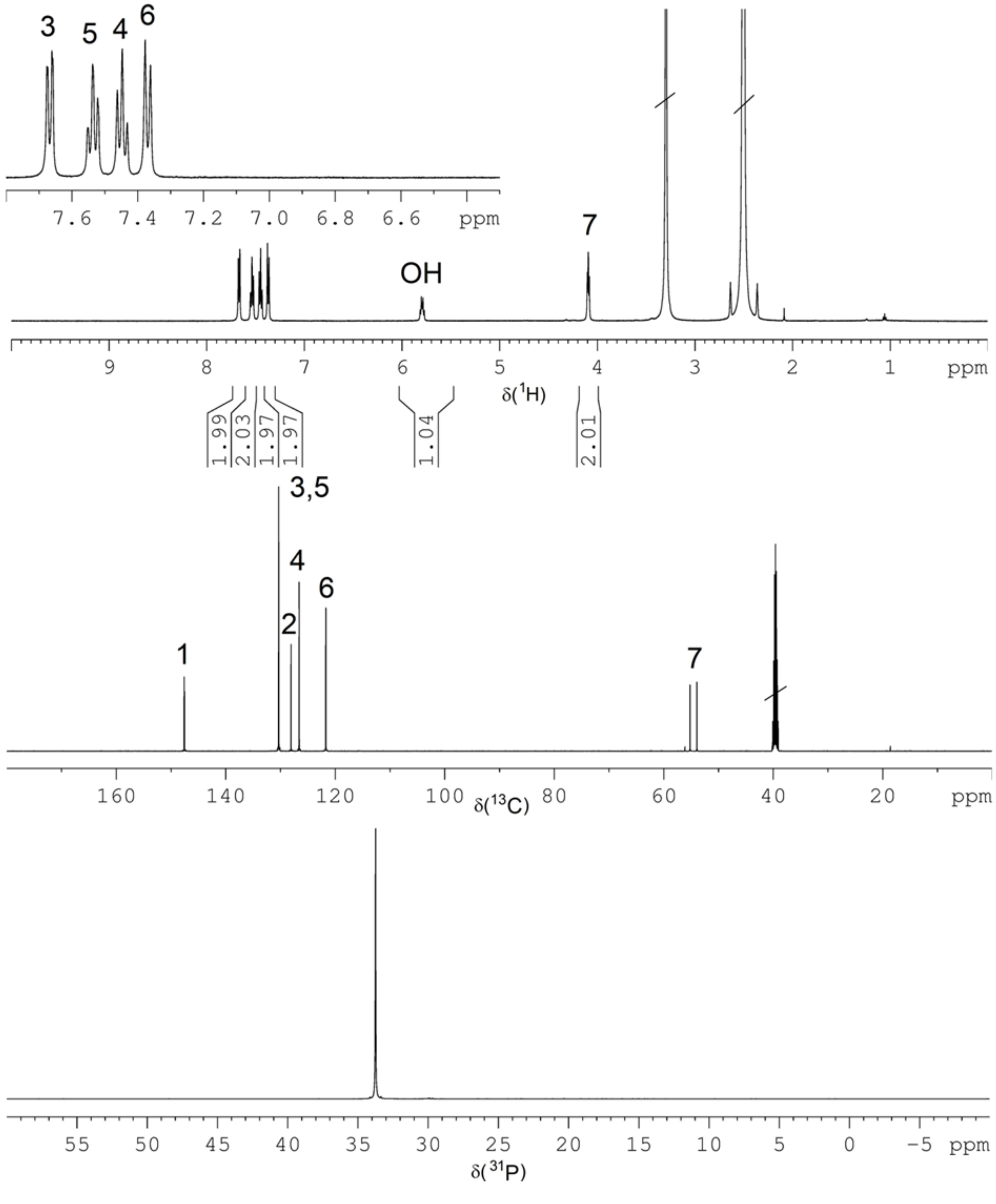

Figure S14. ^{1}H, ^{13}C and ^{31}P NMR spectrum of **FA-BPPO** (solvent: DMSO-d_6).

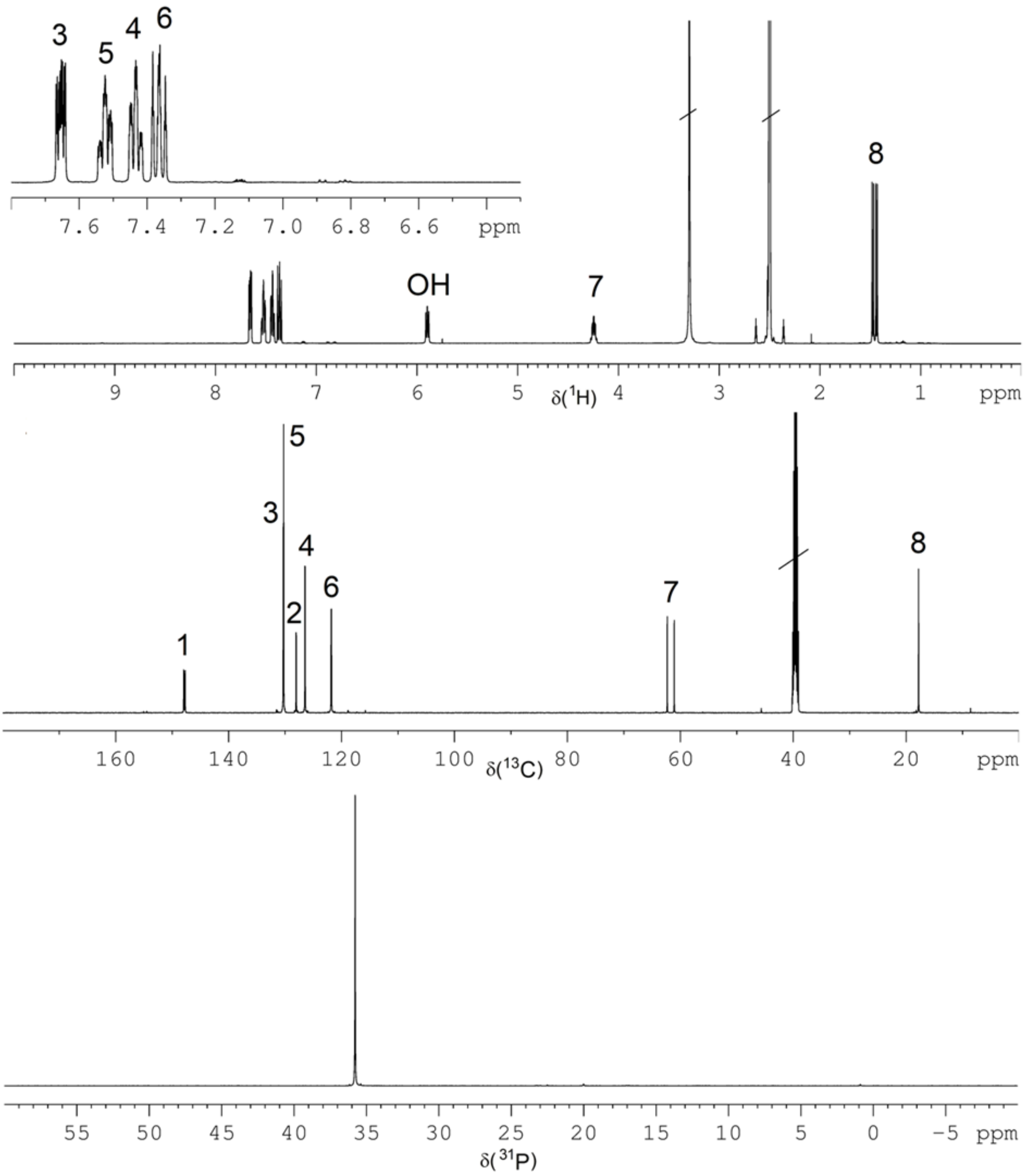

Figure S15. ^{1}H, ^{13}C and ^{31}P NMR spectrum of **AA-BPPO** (solvent: DMSO-d_6).

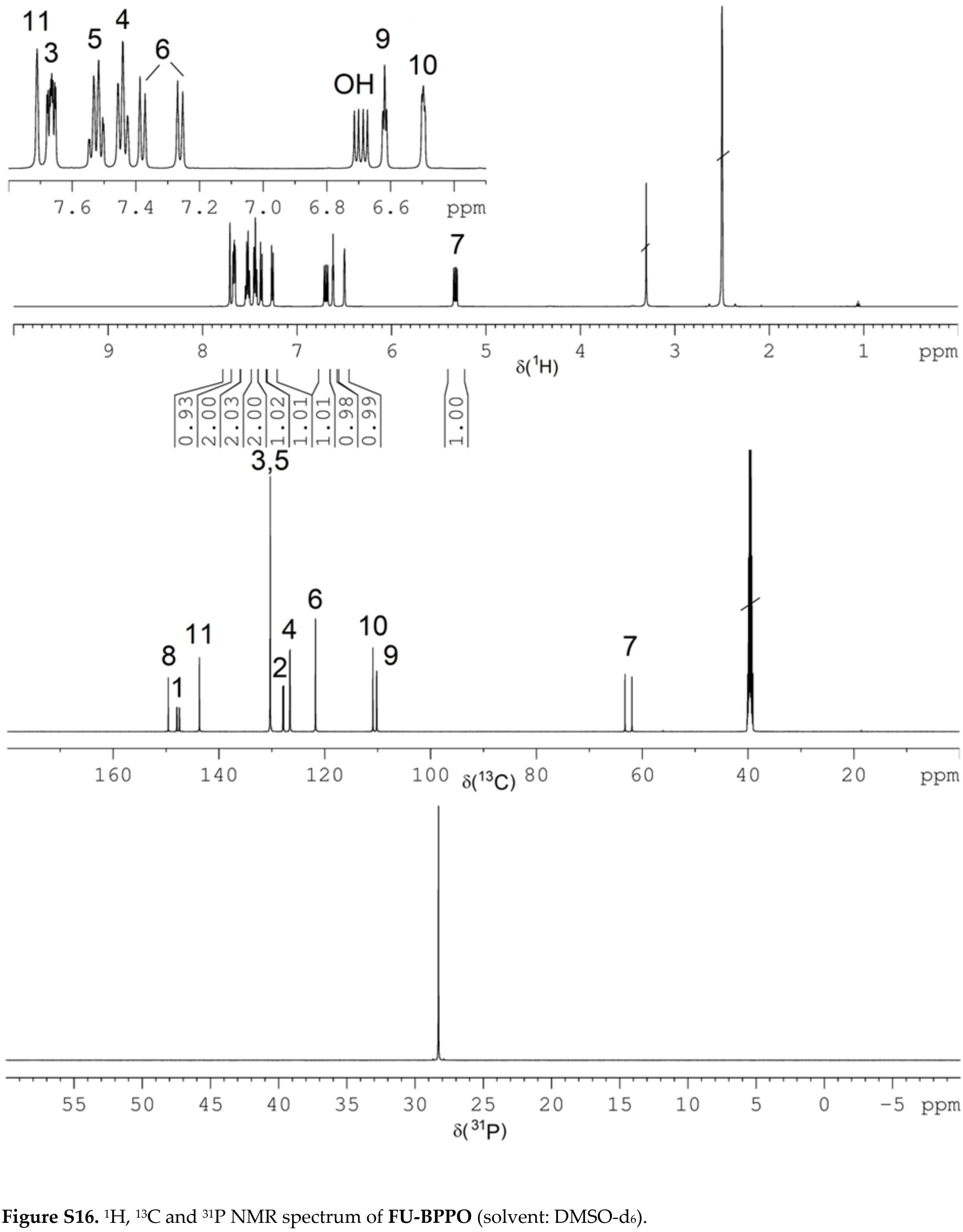

Figure S16. ^{1}H, ^{13}C and ^{31}P NMR spectrum of **FU-BPPO** (solvent: DMSO-d_6).

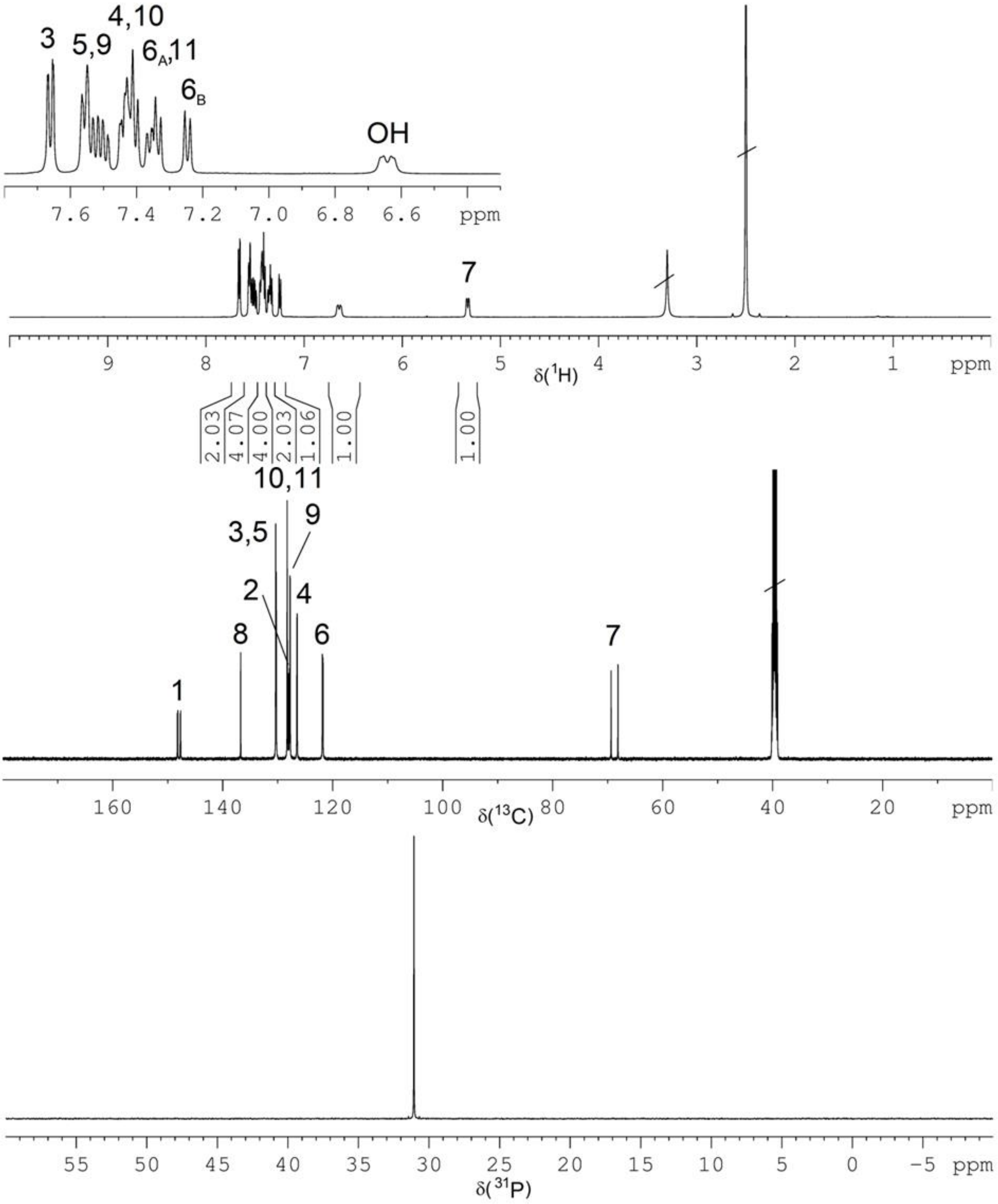

Figure S17. ^{1}H, ^{13}C and ^{31}P NMR spectrum of **BA-BPPO** (solvent: DMSO-d_6).

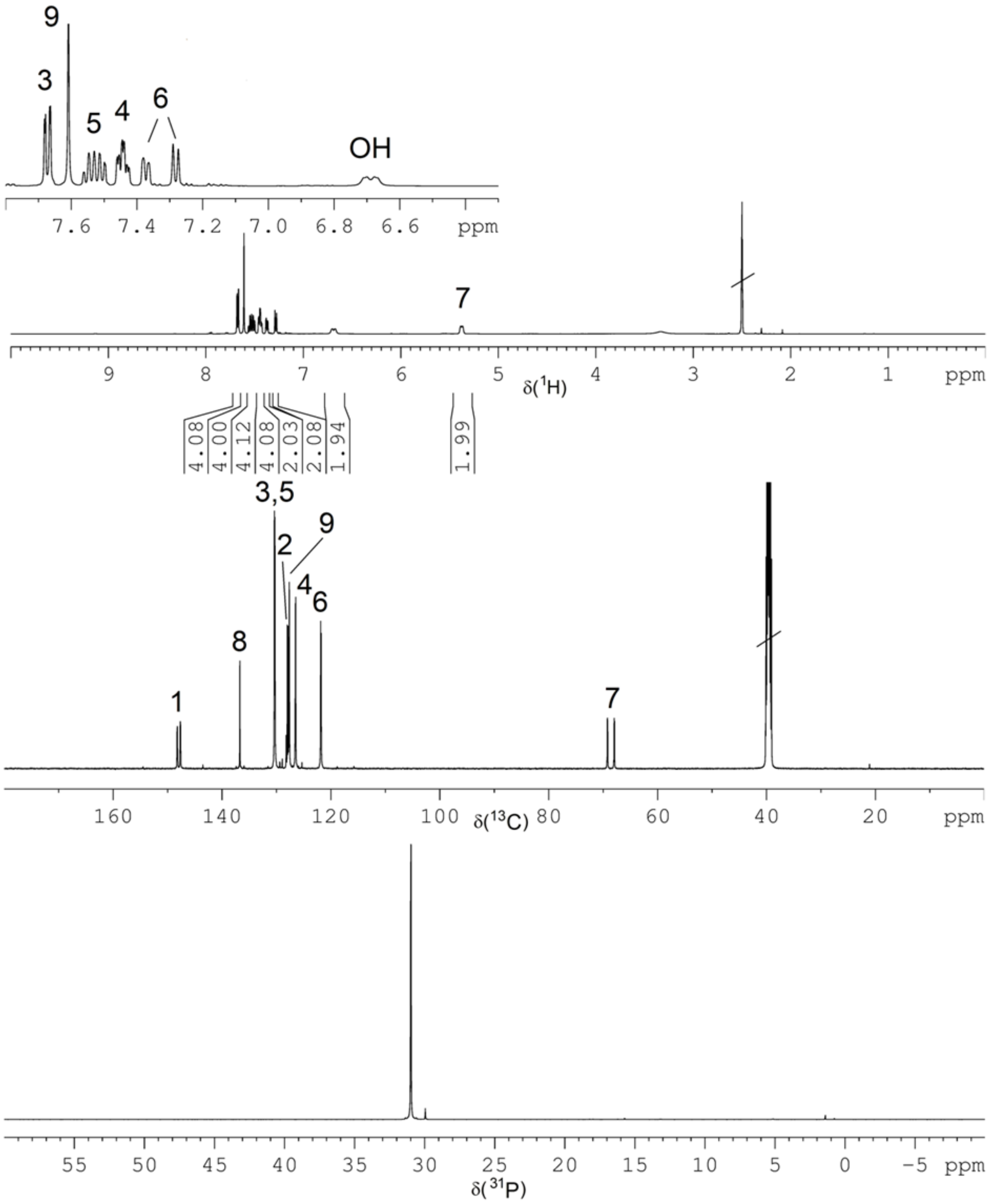

Figure S18. ^{1}H, ^{13}C and ^{31}P NMR spectrum of **TA-BPPO** (solvent: DMSO-d_6).

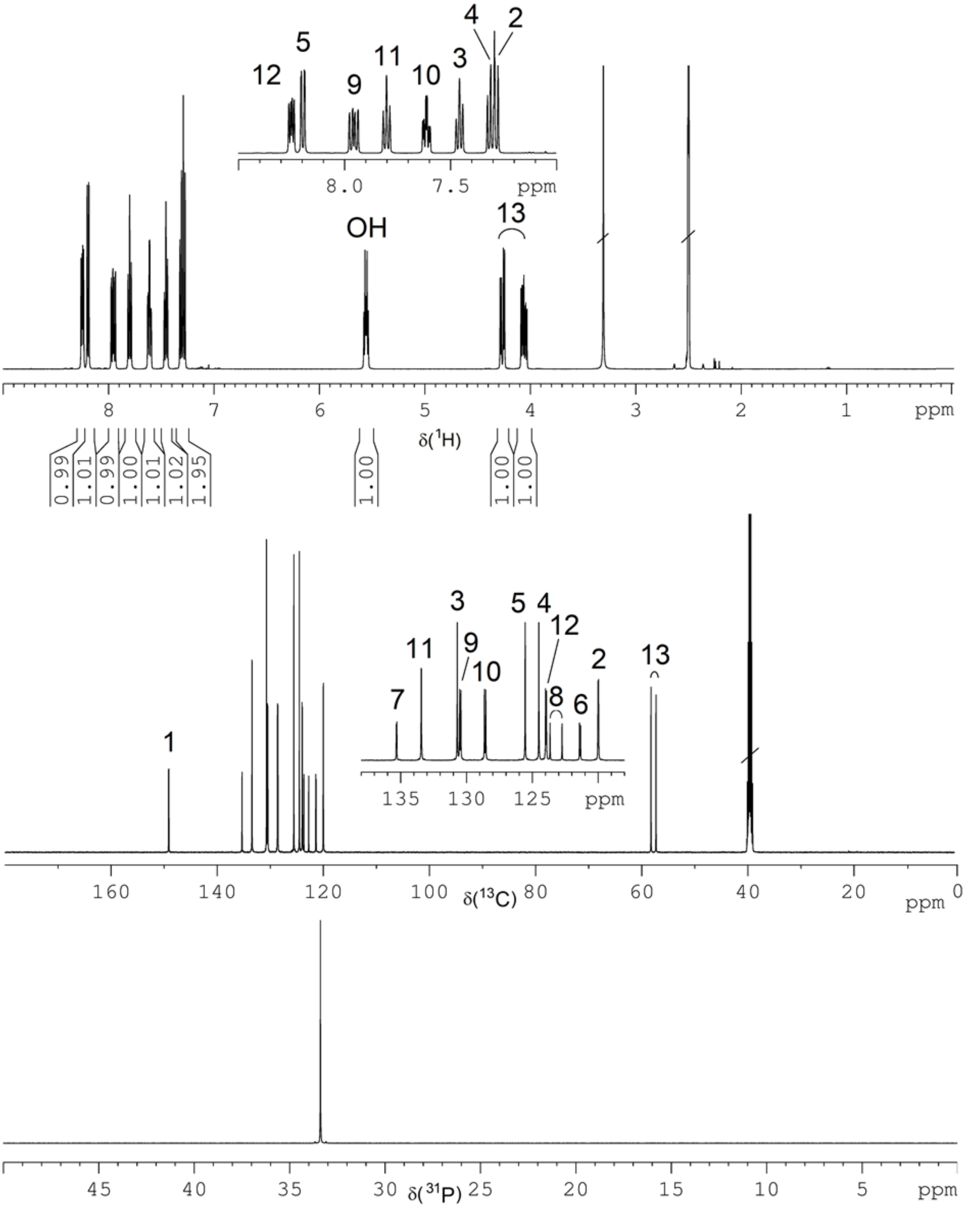

Figure S19. ^{1}H, ^{13}C and ^{31}P NMR spectrum of **FA-DOPO** (solvent: DMSO-d_6).

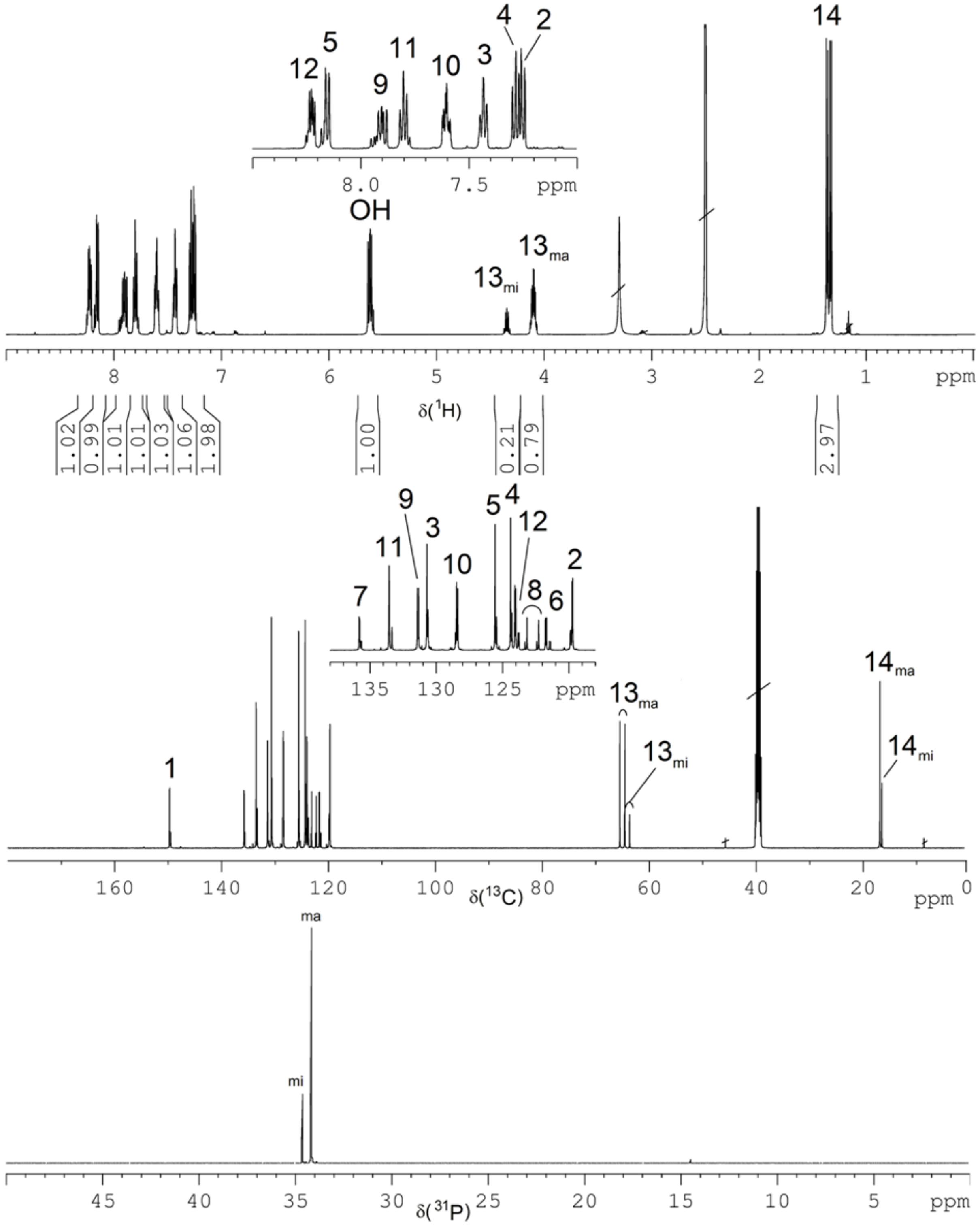

Figure S20. ^{1}H, ^{13}C and ^{31}P NMR spectrum of **AA-DOPO** (solvent: DMSO-d_6).

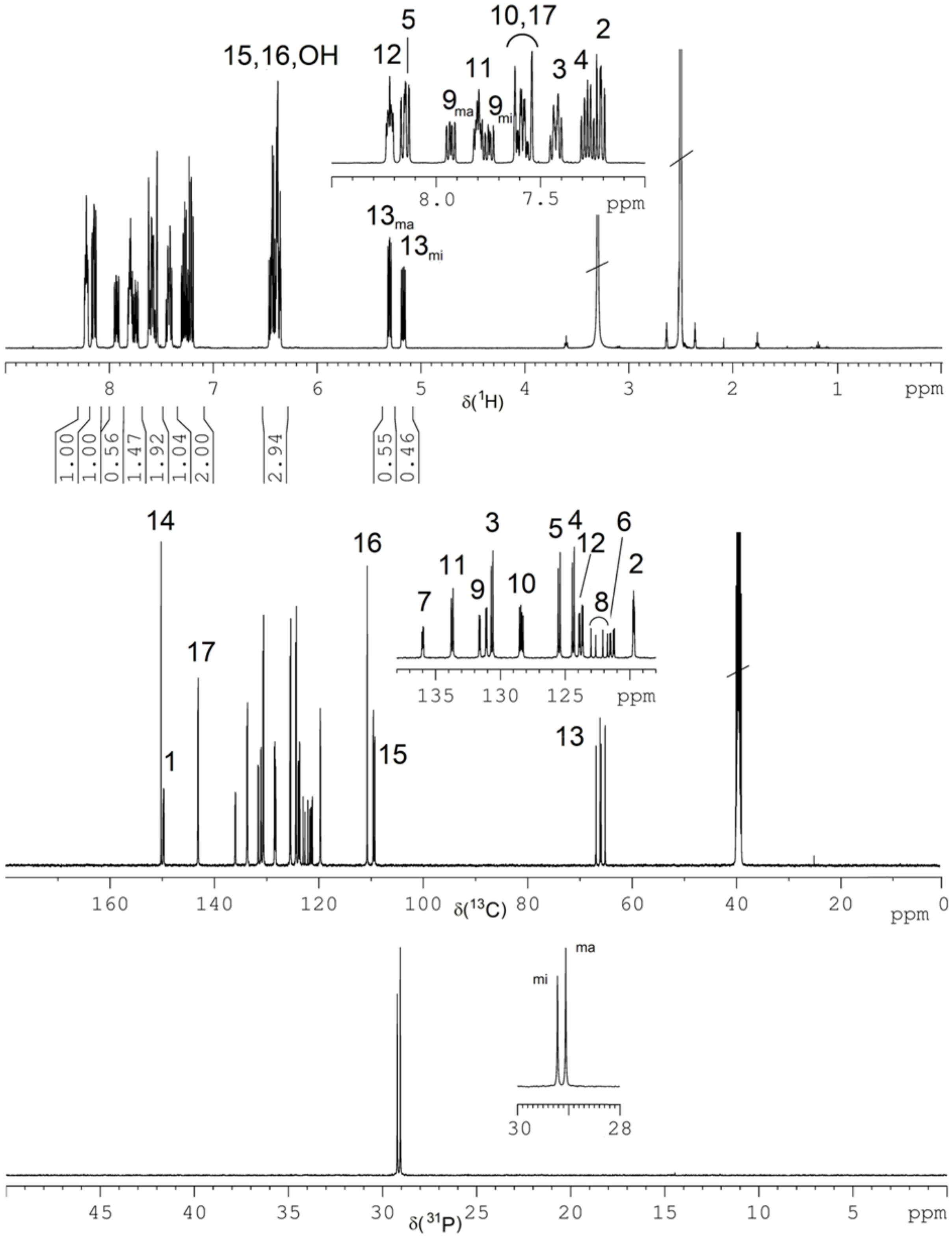

Figure S21. ^{1}H, ^{13}C and ^{31}P NMR spectrum of **FU-DOPO** (solvent: DMSO-d_6).

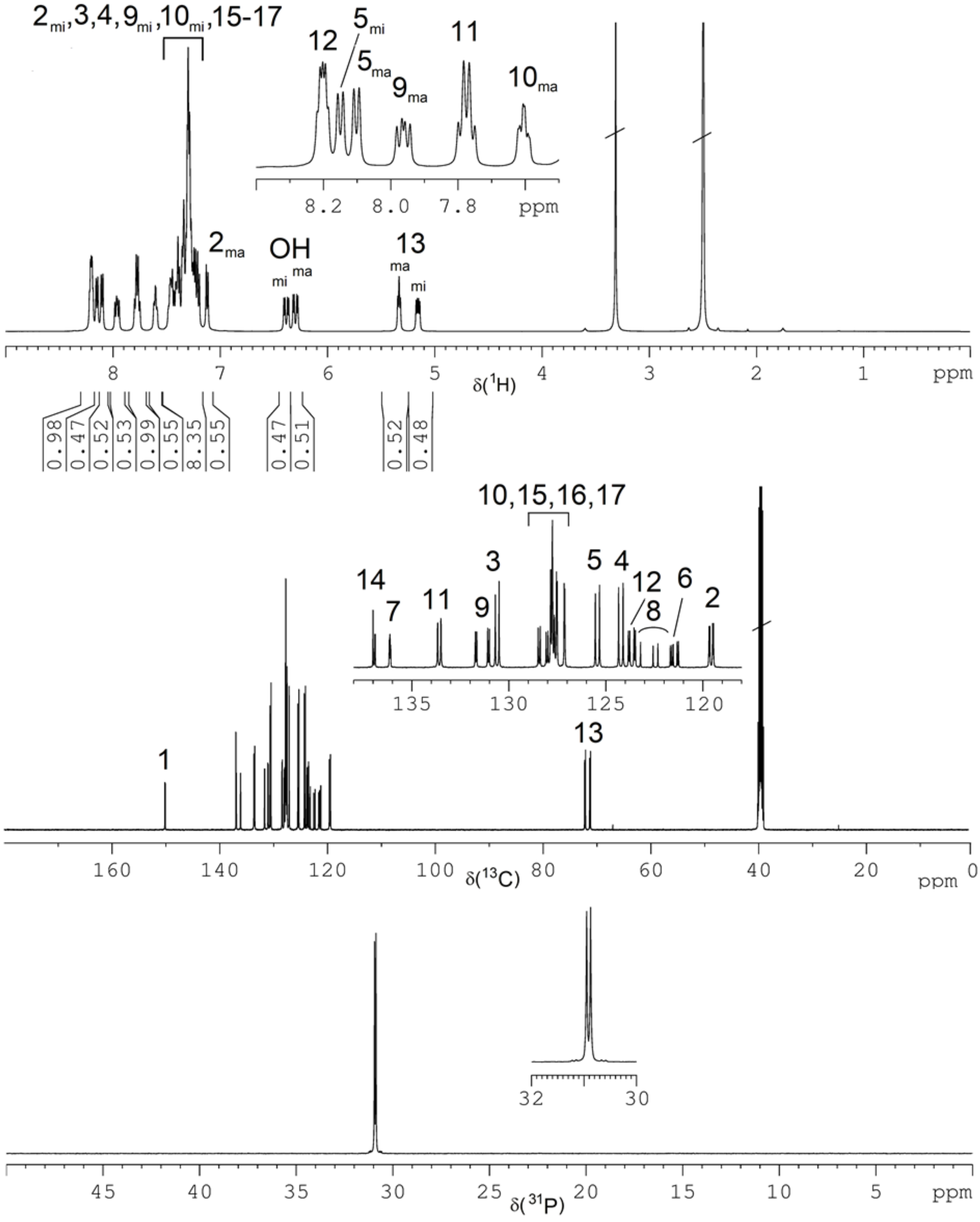

Figure S22. ^{1}H, ^{13}C and ^{31}P NMR spectrum of **BA-DOPO** (solvent: DMSO-d_6).

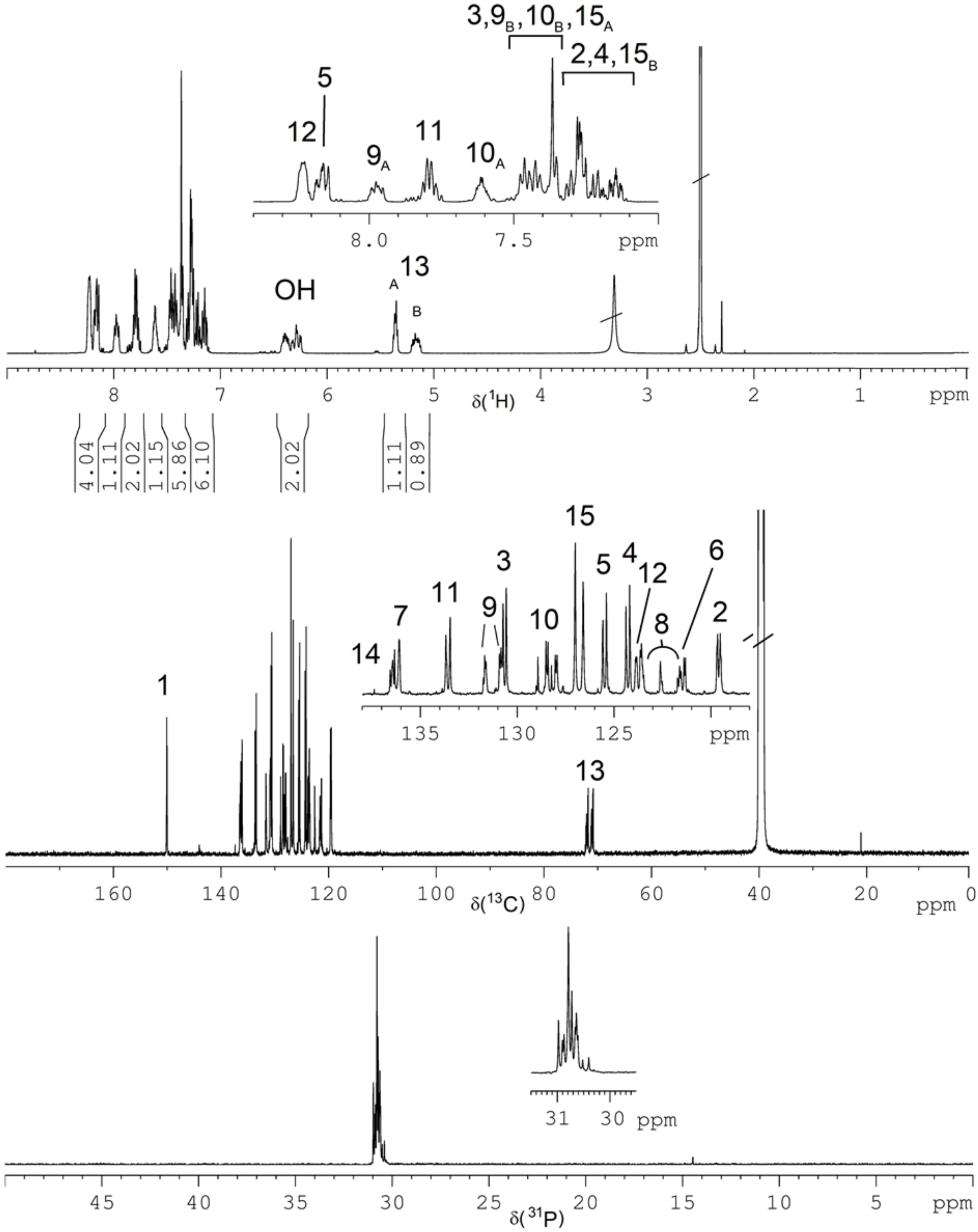

Figure S23. ^1H, ^{13}C and ^{31}P NMR spectrum of **TA-DOPO** (solvent: DMSO-d_6).

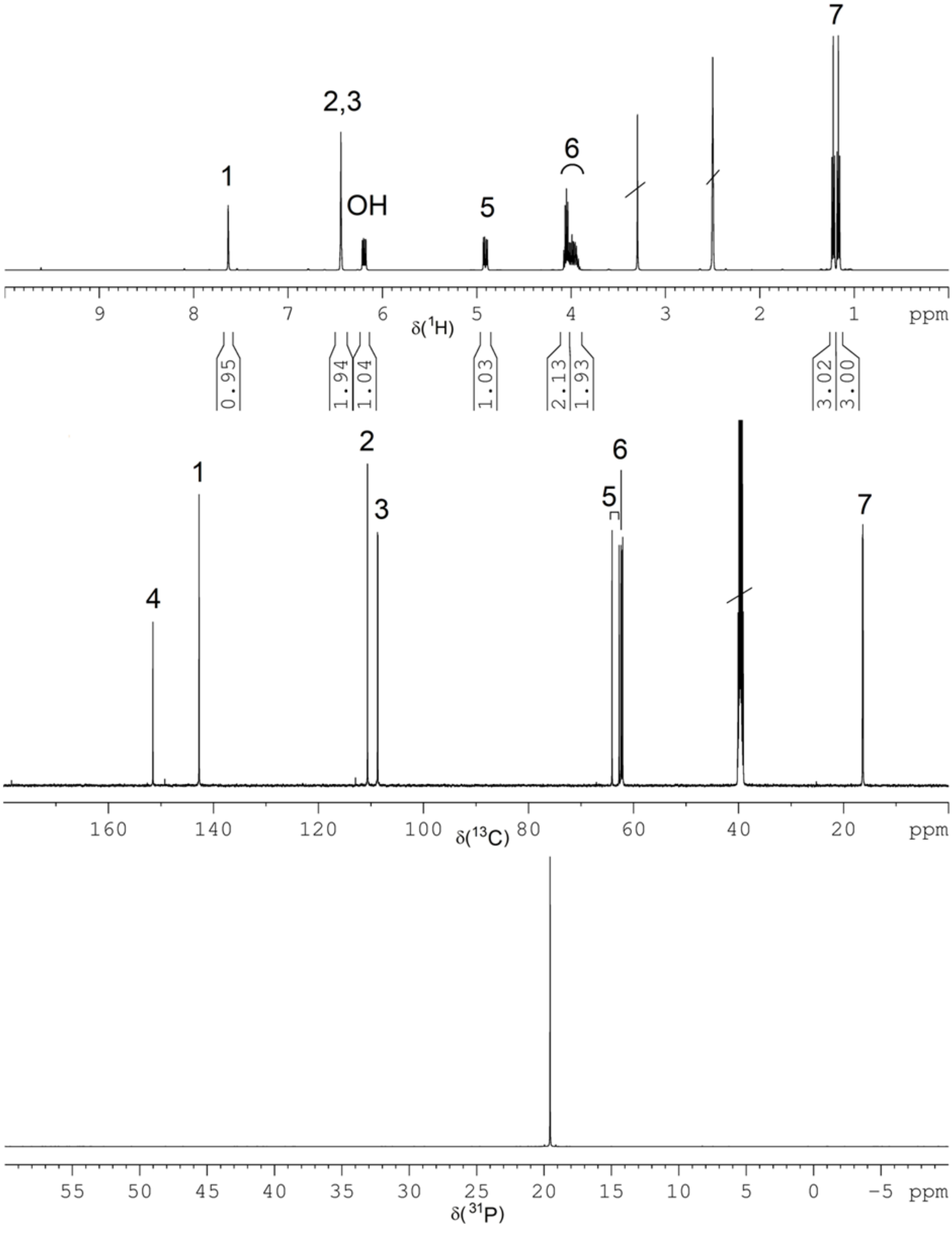

Figure S24. ^{1}H, ^{13}C and ^{31}P NMR spectrum of **FU-EP** (solvent: DMSO-d_6).

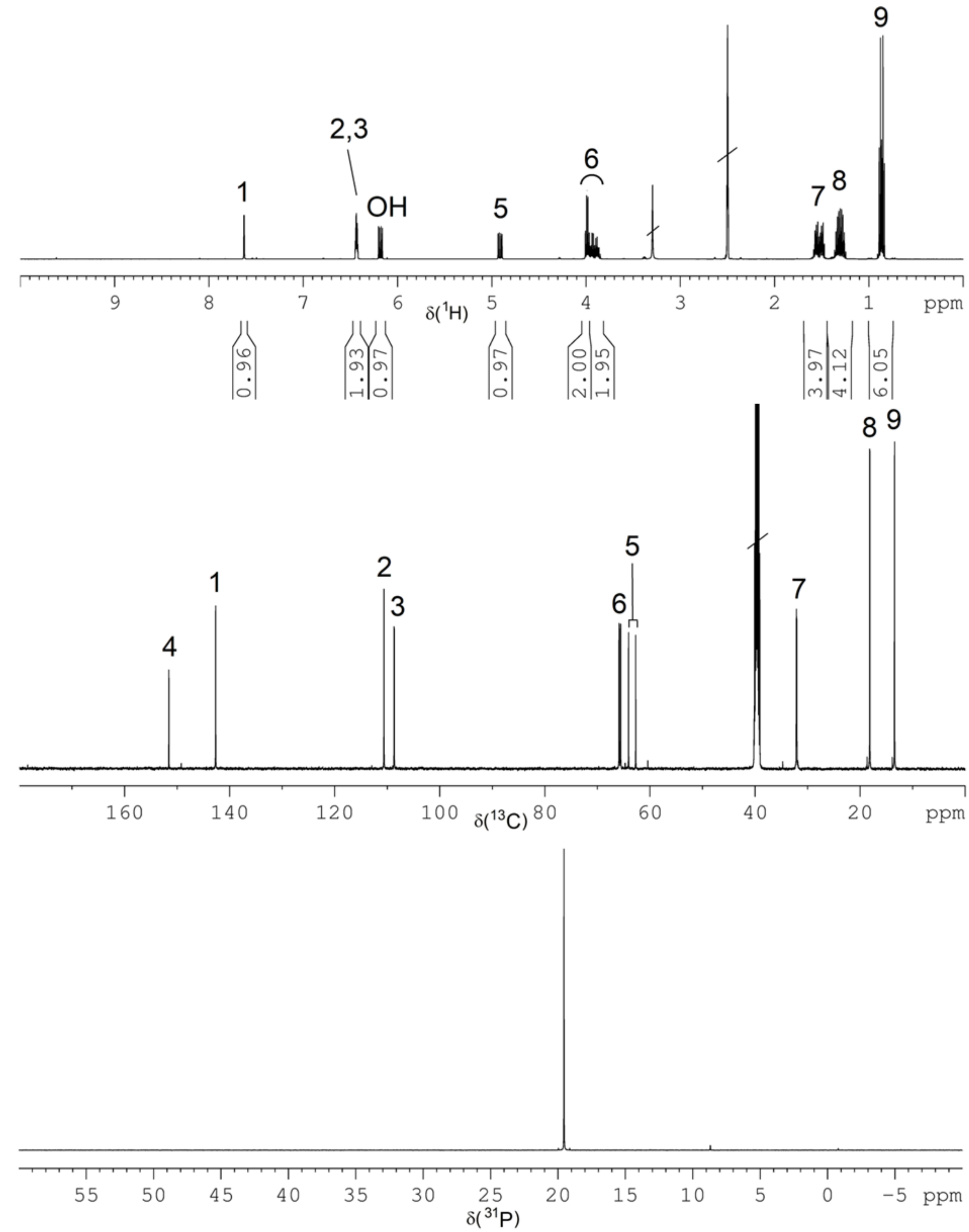

Figure S25. ^{1}H, ^{13}C and ^{31}P NMR spectrum of **FU-BP** (solvent: DMSO-d_6).

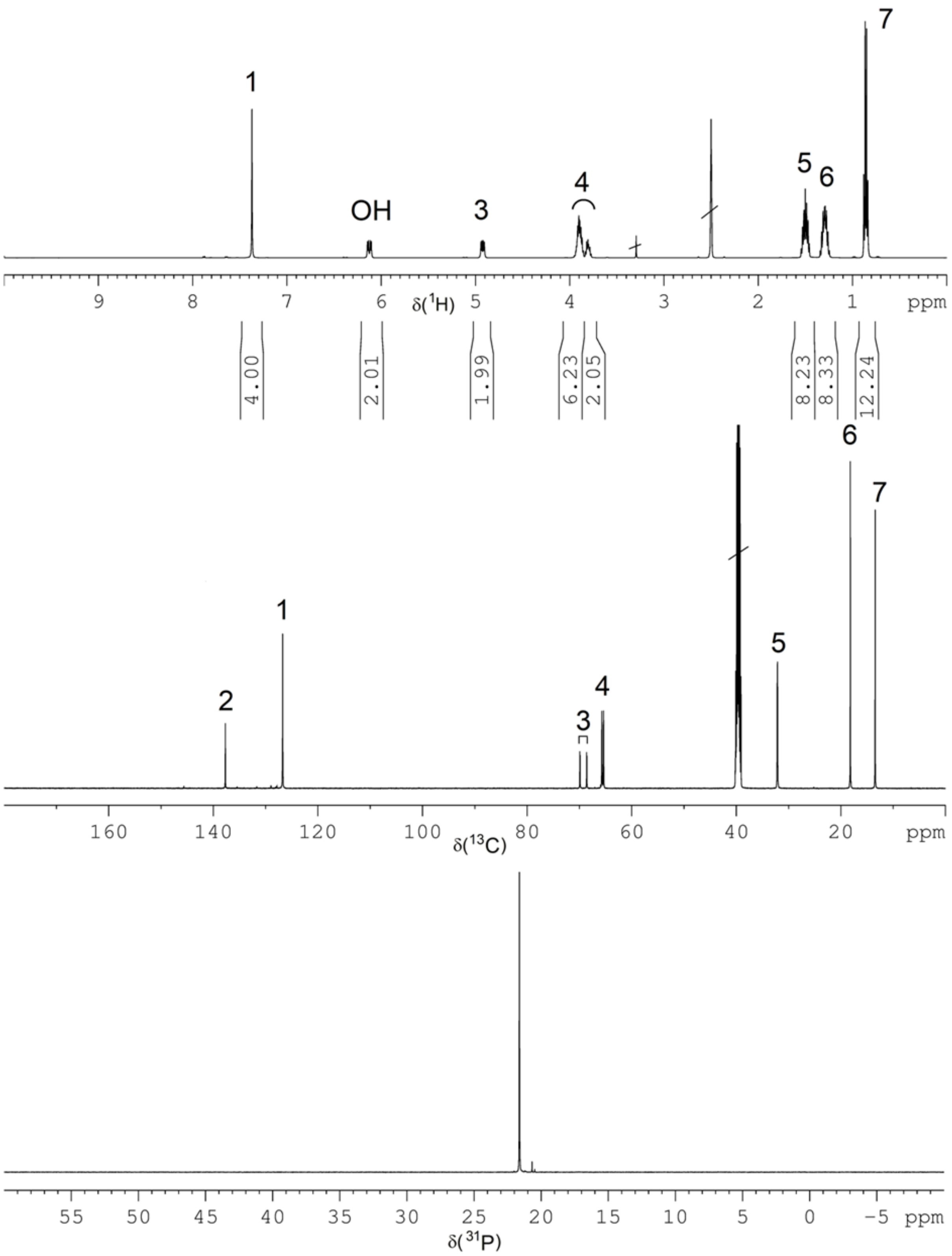

Figure S26. ^{1}H, ^{13}C and ^{31}P NMR spectrum of **TA-BP** (solvent: DMSO-d_6).

3. PIR Foam compositions studied

Table S1. PIR foam compositions studied.

Foam	TEGOSTAB B8421	Emulsogen TS100	TEP	PEG 400	Catalyst	PEP50AD	Pentane	Desmodur 44V70L	Mass of FR
	[g]	[g]	[g]	[g]	[g]	[g]	[g]	[g]	[g]
FA-BP/TEP-1.0P	4.0	2.0	5.0	16.0	2.5	53.0	15.0	151.8	13.0
FA-BP-0.7P	4.0	2.0	-	16.0	2.5	53.0	15.0	151.8	13.0
FA-BPPO/TEP-1.0P	4.0	2.0	5.0	16.0	2.5	53.0	15.0	151.8	15.2
FA-BPPO-0.7P	4.0	2.0	-	16.0	2.5	53.0	15.0	151.8	15.2
AA-BPPO/TEP-1.0P	4.0	2.0	5.0	16.0	2.5	53.0	15.0	151.8	16.0
FU-BPPO/TEP-1.0P	4.0	2.0	5.0	16.0	2.5	53.0	15.0	151.8	19.5
FU-BPPO-0.7P	4.0	2.0	-	16.0	2.5	53.0	15.0	151.8	19.5
BA-BPPO/TEP-1.0P	4.0	2.0	5.0	16.0	2.5	53.0	15.0	151.8	20.0
BA-BPPO-0.7P	4.0	2.0	-	16.0	2.5	53.0	15.0	151.8	20.0
TA-BPPO/TEP-1.0P	4.0	2.0	5.0	16.0	2.5	53.0	15.0	151.8	17.5
TA-BPPO-0.7P	4.0	2.0	-	16.0	2.5	53.0	15.0	151.8	17.5
FA-DOPO/TEP-1.0P	4.0	2.0	5.0	16.0	2.5	53.0	15.0	151.8	14.2
FA-BPPO-0.7P	4.0	2.0	-	16.0	2.5	53.0	15.0	151.8	15.2
AA-DOPO/TEP-1.0P	4.0	2.0	5.0	16.0	2.5	53.0	15.0	151.8	15.0
FU-DOPO/TEP-1.0P	4.0	2.0	5.0	16.0	2.5	53.0	15.0	151.8	18.5
FU-DOPO-0.7P	4.0	2.0	-	16.0	2.5	53.0	15.0	151.8	18.5
BA-DOPO/TEP-1.0P	4.0	2.0	5.0	16.0	2.5	53.0	15.0	151.8	19.0
BA-DOPO-0.7P	4.0	2.0	-	16.0	2.5	53.0	15.0	151.8	19.0
TA-DOPO/TEP-1.0P	4.0	2.0	5.0	16.0	2.5	53.0	15.0	151.8	16.5
TA-DOPO-0.7P	4.0	2.0		16.0	2.5	53.0	15.0	151.8	16.5
FU-EP-0.7P	4.0	2.0		16.0	2.5	53.0	15.0	151.8	13.5
FU-EP/TEP-1.0P	4.0	2.0	5.0	16.0	2.5	53.0	15.0	151.8	13.5
FU-BP-0.7P	4.0	2.0		16.0	2.5	53.0	15.0	151.8	17.0
FU-BP/TEP-1.0P	4.0	2.0	5.0	16.0	2.5	53.0	15.0	151.8	17.0
TA-BP-0.7P	4.0	2.0		16.0	2.5	53.0	15.0	151.8	15.0
TA-BP/TEP-1.0P	4.0	2.0	5.0	16.0	2.5	53.0	15.0	151.8	15.0

4. Compression strength of PIR foams with phospha-aldol adducts

Table S2. Compression strengths of the PIR foams with phospha-aldol adducts.

PIR Foam	F_{max} [kPa]
FA-BP-0.7P	240
FA-BP/TEP-1.0P	258
FA-BPPO-0.7P	246
FA-BPPO/TEP-1.0P	337
AA-BPPO/TEP-1.0P	250
FU-BPPO-0.7P	290
FU-BPPO/TEP-1.0P	297
BA-BPPO-0.7P	263
BA-BPPO/TEP-1.0P	267
TA-BPPO-0.7P	250
TA-BPPO/TEP-1.0P	390
FA-DOPO/TEP-1.0P	219
AA-DOPO/TEP-1.0P	328
FU-DOPO-0.7P	322
FU-DOPO/TEP-1.0P	252
BA-DOPO-0.7P	250
BA-DOPO/TEP-1.0P	257
TA-DOPO-0.7P	254
TA-DOPO/TEP-1.0P	224
FU-EP-0.7P	226
FU-EP/TEP-1.0P	275
FU-BP-0.7P	285
FU-BP/TEP-1.0P	277
TA-BP-0.7P	320
TA-BP/TEP-1.0P	356

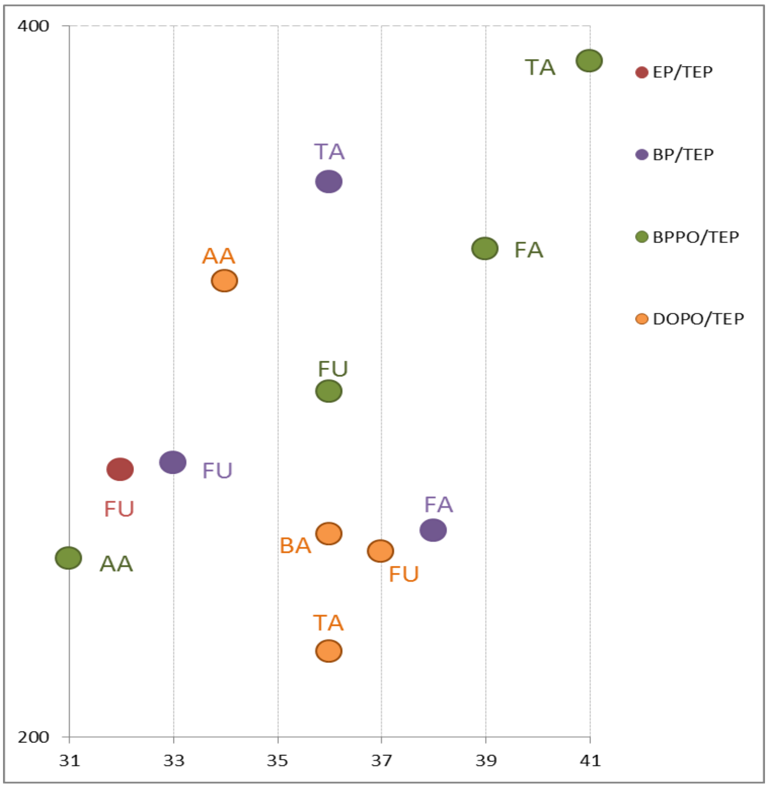

Figure S27. Dependence of the compression strength on the density of selected IR foams with different FR additives.

5. Thermal decomposition of the PIR foams studied by TGA

Table S3. TGA data of the PIR foams studied (TGA under nitrogen with a scan rate of 10 K/min).

**FR additive/ foam name-P content [wt.-%]**	**Decomposition step $T_{max,dec}$//Mass loss at $T_{max,dec}$ [°C]//[wt.-%]**	**Residue at 800 °C [wt.-%]**
0 (PIR-0)	**1. 326**	26.6
TEP-0.3	1. 219//14.9<br>**2. S285/325//36.5**<br>3. 405/S463//20.3<br>4. 575//5.2	21.1
TPP-0.7	1. 225//9.1<br>**2. 332//38.3**<br>3. 395//9.3<br>4. S490/S480//9.0	29.3
TPP/TEP-1.0	**1. 280/340//39.2**<br>2. 410/S460//21.1<br>3. S560//9.6	29.8
EA-BPPO-0.7	1. S150/S280/**330**//46.4<br>2. 410/S480/S575//26.7	24.6
EA-BPPO/TEP-1.0	**1. 321**	22.6
PA-BPPO/TEP-1.0	**1. 320**	34.6
DMI-BPPO/TEP-1.0	1. 180//5.3<br>2. 285//16.0<br>**3. 329//27.2**<br>4. 422//10.4<br>5. 465//9.7	23.3
DMI-DOPO/TEP-1.0	1. 175//4.2<br>**2. S290/332//39.7**<br>**3. 427/S490/S570//29.7**	23.9
FA-BPPO-0.7	**1. S250/S295/316//35.6**<br>2. 403//17.1<br>3. 524/s570//12.3	29.7
FA-BPPO/TEP-1.0	**1. S262/S295/317//35.8**<br>2. 381//21.1<br>3. 515//7.7	29.0

BA-BPPO-0.7	1. 242//8.0<br>**2. S295/315//26.1**<br>3. 500//15.3<br>4. 515//15.1	31.4
BA-BPPO/TEP-1.0	1. S185//3.2<br>2. 245//7.8<br>**3. S300/317//25.5**<br>4. 400//14.6<br>5. 525//15.2	30.8
BA-DOPO-0.7	1. S150/190//2.6<br>2. S235//4.2<br>**3. S290/320//26.5**<br>**4. S375/425//31.3**<br>5. S545//7.8	25.4
BA-DOPO/TEP-1.0	1. S130/192//3.4<br>2. S240//4.3<br>**3. S295/320//26.2**<br>**4. 427//30.1**<br>5. S550//8.2	25.0
FU-BPPO-0.7	1. 185//1.8<br>2. 240//3.5<br>**3. S300/320//29.7**<br>4. 404//11.8<br>5. 522//16.3	34.0
FU-BPPO/TEP-1.0	1. S125/185//1.8<br>2. 240//3.5<br>3. S300/320//29.7<br>4. 405//11.8<br>5. 522//16.3	34.6
FU-DOPO-0.7	1. 190//2.4<br>2. S235/S295/320//30.6<br>3. 423//30.8<br>4. S550//8.2	25.4
FU-DOPO/TEP-1.0	1. 192//2.5<br>2. S240//4.2<br>**3. S300/322//26.5**<br>4. 418//29.1<br>5. S555//8.3	25.6
TA-BPPO-0.7	1. 233/6.1<br>**2. 325/29.3**<br>3. 405/11.8<br>4. 515/17.8	31.5

TA-BPPO/TEP-1.0P	1. 235//6.3<br>**2. S300/320//29.4**<br>3. 404//11.8<br>4. 518//17.5	32.9
TA-DOPO-0.7P	1. 232/4.1<br>**2. 322/29.5**<br>**3. 422/S540/37.1**	26.7
TA-DOPO/TEP-1.0P	1. 240//6.7<br>**2. S295/322//25.6**<br>**3. 431//30.2**<br>4. S550//8.7	
TA-BP-0.7P	1. 236//9.1<br>**2. S275/324//31.9**<br>3. 420//10.1<br>4. 470/S535//17.8	27.8
TA-BP/TEP-1.0P	1. 232//7.1<br>**2. S295/324//31.9**<br>3. 422/S475//21.7<br>4. S560//5.6	27.4
FU-BP-0.7	1. S116//7.6<br>2. 230//26.4<br>3. S293//8.5<br>**4. 319//19.7**<br>5. 413//3.6	33.7
FU-BP/TEP-1.0	1. 228//9.0<br>2. 292//5.2<br>**3. 318//10.1**<br>**4. S391//17.3**<br>5. 412//2.3	34.5
FA-BP-0.7	1. 185//4.8<br>**2. S240/325//37.5**<br>3. 415//10.4<br>4. 465/S520//16.1	28.9
FA-BP/TEP-1.0	1. S170//1.7<br>**2. S260/321//40.7**<br>3. 410/S465//16.9<br>4. 520//8.4	30.3

BA: benzaldehyde; BP: butylphosphonate; BuA: butylacrylate; EA: ethylacrylate; DMI: dimethylitaconate; FA: formaldehyde; FU: furfurylaldehyde; PA: phenyl acrylate; TA: terephthalaldehyde.
S: shoulder
Bold black: main decomposition maximum caused by PIR decomposition
Bold blue: main decomposition maximum caused by DOPO decomposition

Table S4. Summary of pyrolysis-GC/MS data obtained for PIR foams with phospha-aldol adducts. Results with phospha-Michael adducts have been published before in. Selected samples are given for comparison.

PIR foam	T [°C]	TEP	DBM	Decanal	Bu-OH	DEG	DBP	Dioxane	EG-vinyl-ether	PA/ PAA deriv.	MDI	TEG	2,2′-BP diol	Aniline deriv.	Glycol	Furfural	TBP
Retention time [min]		8.41	7.55	9.47	2.37	6.25	11.02	2.72	2.85	10.87<br>16.59	18.24	9.71<br>10.55	14.49	6.48<br>7.70<br>7.82<br>8.55	2.56	4.24<br>4.48	14.27
PIR-0	327					+								+			
	468					+				+				+			
TPP/TEP-1.0	215	+															
	324					+											+
	465					+								+			
EA-BPPO/TEP-1.0	130	+															
	245	+															
	465									+				+			
FA-BP/TEP-1.0	179	+															
	324				+	+	+			+							
	465					+		+	+	+		+					
FA-BPPO-0.7	179	+	+														
	324				+		+			+							+
	465							+						+			
FA-BPPO/TEP-1.0	295	+								+							
	381					+		+		+	+	+	+				
	527							+						+			
FU-BPPO/TEP-1.0	183														+		
	321	+	+					+		+					+		
	405							+		+		+	+	+		+	
	522					+		+						+			
FU-DOPO/TEP-1.0	191	+															
	318	+															
	423					+			+			+		+			

TA-BPPO/TEP-1.0	232														+		
	323	+				+				+			+				
	403					+		+					+				
	518					+				+			+	+		+	
TA-DOPO/TEP-1.0	191	+													+	+	
	318	+			+	^+				+						+	
	423				+		+	+		+		+		+	+		
TA-BP/TEP-1.0	236	+					+			+							+
	322	+			+		+			+							+
	477							+		+				+			
FU-BP/TEP-1.0	230	+					+	+		+						+	+
	318	+			+			+								+	
	412					+		+						+			
	534					+		+						+			

+

Py-GC/MS: TEP: triethylphosphate; DBM: dibutoxymethane; Bu-OH: butanol; DEG: diethylene glycol; DBP: dibutyphosphite; EG-vinylether: ethylene glycol vinyl ether; PAA: phthalic acid or phthalic anhydride; MDI: m,ethylenediisicyanate; TEG: triethylene glycol; 2,‘-BP: 2,2‘-biphenyl; TBP: tributylphosphate.

Pyrolyzer Pyroprobe 5000 (CDS Analytical, Inc., Oxford, PA, USA) with platinum filament, interface CDS1500 (CDS Analytical, Inc., Oxford, PA, USA), GC7890A (Agilent Technologies, Santa Clara, Ca, USA), with an inlet temperature of 280 °C, an oven temperature of 50 °C/2 min, 12 K/min to 280 °C, GC column HP-5MS (30 cm·250 cm innedr diameter and 0.25 µm layer thickness; gas flow: helium, 1 mL/min; mass specxtrometry: Agilent MSD (mass-selective detector) 5975C inert XL EI/CI (Agilent Technologies, Santa Clara, Ca, USA) at 70 eV iun the scan range of 15–550).

6 Cone calorimeter results: MARHE vs. char plots

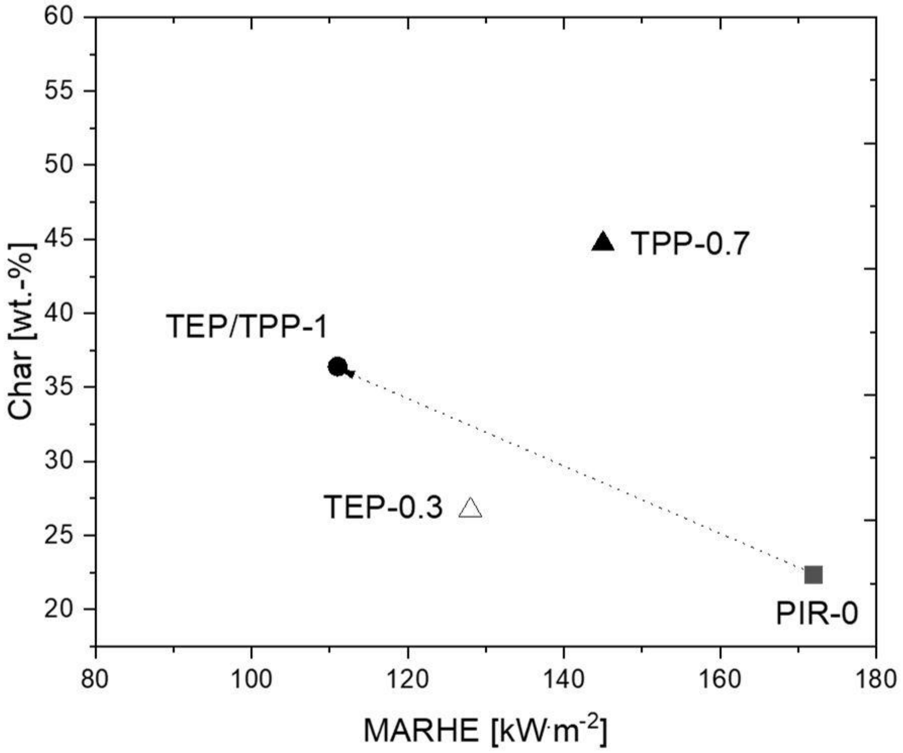

Figure S28. MARHE vs. char plots for the control and state-of-the-art PIR foams studied.

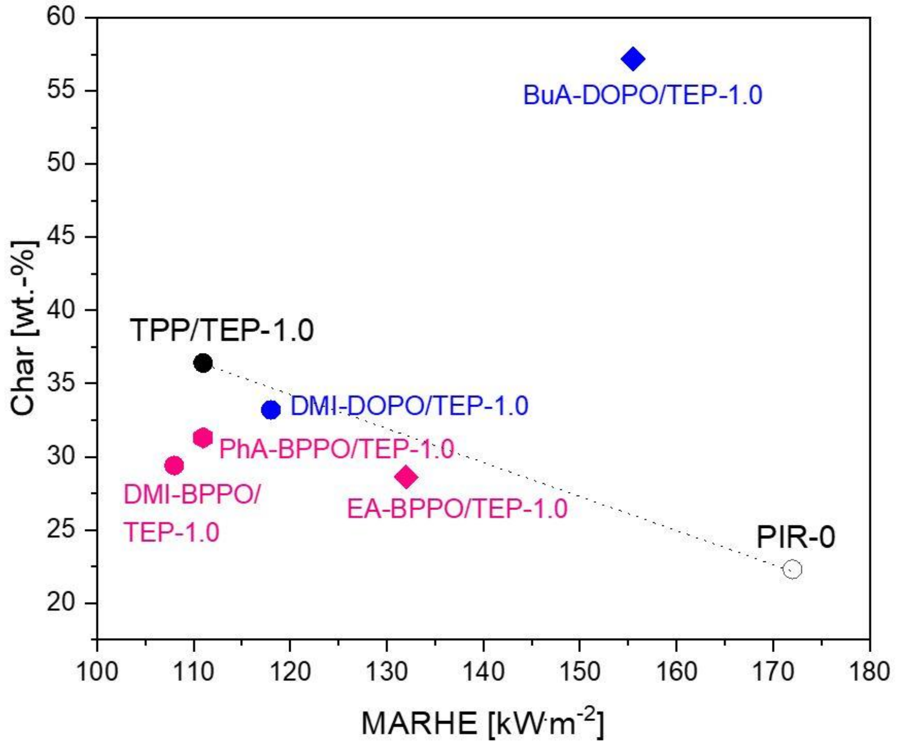

Figure S29. MARHE vs. residue plot for PIR foams with non-reactive BPPO additives (PhA: phenylacrylate; DMI: dimethylitaconate; EA: ethylacrylate, BuA: butylacrylate).

Reference

1 J. Lenz, D. Pospiech, M. Paven, R. W. Albach, M. Günther, B. Schartel and B. Voit. Improving the flame retardance of polyisocyanurate foams by dibenzo [d, f][1, 3, 2] dioxaphosphepine 6-oxide-containing additives. *Polymers* **2019**, ***11***, 1242.
